# Hepatitis Associated with *Catha edulis* Consumption—A Single-Center Study

**DOI:** 10.3390/jcm14041206

**Published:** 2025-02-12

**Authors:** Ali Someili, Mostafa Mohrag, Mohammed Abdulrasak

**Affiliations:** 1 Department of Medicine, Faculty of Medicine, Jazan University, Jazan 45142, Saudi Arabia; mmohrag@jazanu.edu.sa; 2 Department of Clinical Sciences, Lund University, 22100 Malmo, Sweden; mohammed.abdulrasak@med.lu.se; 3 Department of Gastroenterology and Nutrition, Skane University Hospital, 21428 Malmo, Sweden

**Keywords:** Khat, liver injury, drug-induced liver injury (DILI), autoimmune hepatitis (AIH), drug-induced autoimmune-like hepatitis (DI-ALH), R-factor

## Abstract

**Background/Objectives:** *Catha edulis*, also known as Khat, is a stimulant with hepatotoxic properties. Studies reporting laboratory patterns are scarce. The aim was to assess the patterns associated with hepatic dysfunction due to Khat usage. **Methods**: Patients with liver injury and self-reported Khat consumption presenting to the gastroenterology department at the King Fahad Central Hospital in Jazan between January 2017–May 2024 were retrospectively included in the study. Patients with any signs of cirrhosis or viral hepatitis were excluded to have a more homogenous inclusion. Normal distribution was not assumed; data were presented as the median (IQR or %). **Results**: Sixty-three patients (of which 62 (98.4%) were male) aged 35 (29–41) years were included in the study. An IgG > 20 g/L was present in 41 (61.5%) patients, and the majority (*n* = 48, 76.2%) had a hepatocellular injury pattern based on an R-factor > 5. Over half of the patients had at least one positive autoantibody(ANA 47.6%; SMA 55.6% and AMA 4.8%), while 57 (90.5%) patients received immunosuppressive therapy. **Conclusions**: Khat-induced liver injury seems to be predominantly AIH-like in nature, given the IgG elevation, hepatocellular injury pattern, and relatively high rate of autoantibody positivity.

## 1. Introduction

*Catha edulis*, also known as Khat, is a leafy plant used as a stimulant native to the populations of the African Horn, Yemen, and southern KSA [1,2]. The plant is consumed through intermittent chewing of a certain amount of unprocessed Khat leaves, with retention of the leaves in the wall of the inner cheek with absorption of the active substance initially at the level of the inner buccal lining and later when saliva enters the small bowels, with the remainder of the absorption occurring at that level [3]. Khat chewing is usually done at social gatherings and is a habit mainly pursued by males [4]. The consumption of the leaves in this manner results in euphoria, increasing agreeableness and self-confidence [5]. The active substances responsible for the effects of Khat include Cathinone and Cathine, both of which are amphetamine-like substances that contribute to the toxicity associated with Khat usage [6]. Legislative bodies in the KSA and multiple other countries have deemed the usage of Khat to be illegal [7]. This is due to its addictive nature and associated injury to multiple organs when used. In our region, the prevalence of Khat usage among males is around 50–60% [8].

One of the main organs affected by Khat is the liver, as reported in the literature. Previous reports suggest a wide array of hepatotoxicity patterns associated with Khat, ranging from mild asymptomatic transaminitis [9] with a nonspecific drug-induced liver injury (DILI) pattern all the way to a severe autoimmune hepatitis-like picture with severe jaundice, requiring liver transplantation [10]. Several mechanisms for this have been postulated, which may include a direct cytotoxic effect—as observed in both cellular and animal models [11,12]. In addition, and in animal models, Khat seems to have an immune-stimulating effect [13], which may contribute to the previously described autoimmune hepatitis-like picture [14,15,16,17,18]. The ensuing hepatitis may even respond to treatment with steroids and azathioprine in a similar manner to autoimmune hepatitis (AIH) [19]. Chronic Khat consumption has even been implicated in the development of chronic liver disease with ensuing cirrhosis [20]. The available reports on Khat-associated hepatic injury are, however, scarce and limited mainly to smaller case reports and case series.

In this study, we aim to assess the patterns associated with hepatic dysfunction due to Khat usage in a single-center setting in our region of KSA, with a specific focus on the laboratory results obtained when the patients were first investigated for their Khat-associated liver injury.

## 2. Materials and Methods

Patients with hepatic dysfunction with self-reported Khat usage presenting to the gastroenterology department at the King Fahad Central Hospital in Jazan were retrospectively included in this study. The time period was January 2017–May 2024. Patients who had established cirrhosis, underlying viral hepatitis, or another cause for hepatic disease (e.g., autoimmune hepatitis (AIH), alcoholic liver disease, metabolic dysfunction-associated steatotic liver disease) were excluded. The data collection was aimed at collecting background data such as age, gender, smoking habits, and underlying alcohol consumption alongside Khat consumption. Those laboratory values that were relevant were collected, including alanine transaminase (ALT), aspartate transaminase (AST), γ-glutamyl transferase (GGT), and alkaline phosphatase (ALP), alongside total bilirubin, normalized international ratio (INR), and albumin. The laboratory results either represented the highest values during admission or the values obtained during outpatient visits. All patients included had an ultrasound study performed on the liver to exclude the presence of imaging signs of cirrhosis. This was performed by consultants in radiology at the local radiology department.

Given the multiple reports suggesting a potential role for Khat in inducing AIH, immunological data associated with that specific diagnosis were collected. These included, if the autoantibodies associated with autoimmune liver disease were present, antinuclear antibodies (ANA), antimitochondrial antibodies (AMA), and smooth muscle antibodies (SMA) alongside LKM1 (liver-kidney microsomal 1) antibodies. Immunoglobulin G levels were also collected, given their association with AIH diagnosis. All patients in this study were offered a liver biopsy. For the patients who agreed to undergo a biopsy, this was noted. However, the biopsy results were not always present, given that biopsies of the liver are interpreted at an outside center, whereby not all biopsy results were available.

There are currently no established guidelines for managing Khat-induced hepatitis. In practice, treatment typically begins with counseling patients on the discontinuation of Khat. Following this, patients are managed similarly to those with autoimmune hepatitis. [21] The treatment regimen starts with prednisone, 40 mg orally daily for 30 days. After this initial phase, and if the thiopurine S-methyltransferase (TPMT)-enzymatic activity is adequate, azathioprine is introduced at a dose of 1–2 mg/kg daily, while prednisone is gradually tapered by 5 mg per week until reaching a maintenance dose of 5–10 mg daily. The treatment process emphasizes active patient involvement, with careful attention given to their preferences regarding the medications used. Additionally, available laboratory values, particularly the transaminase and IgG levels, are thoroughly considered in the treatment decision. In some cases, treatment is withheld, particularly if patients decline it and if the transaminases are not elevated ×2–5 times the upper limit of normal measurements while the bilirubin levels remain normal. In such instances, a close follow-up is ensured.

Comparisons were performed to assess for any potential patterns in the laboratory results of the included patients. This was done based on the AST/ALT ratio > 1, signifying an increased zone III injury, which is associated classically with DILI [22,23]; IgG > 20 g/L, being associated with active/relapsing autoimmune hepatitis; [24] and R-factor, given its usage to determine the pattern of drug-induced liver injury, where an R-factor > 5 suggests hepatocellular injury, 2–5 suggests mixed injury, and <2 suggests purely cholestatic injury [25,26] according to the following formula:

$$R-Factor=\frac{\frac{ALT\;\left(patient\right)}{ALT\;\left(Upperlimit\right)}}{\frac{ALP\;(patient)}{ALP\;(Upperlimit)}}$$

These comparisons were undertaken to determine the dominant laboratory injury pattern in patients with Khat-induced liver injury in detail. The upper limit for ALT in our center was 50 IU/L, and for ALP, 150 IU/L. The R-factor was chosen given its wide usage to assess different DILI patterns in well-renowned databases [27].

Normal distribution was not assumed. The data were represented as the median (interquartile range; IQR) for continuous data and N (%) for discrete data. A Mann–Whitney U test was utilized to assess the differences between the groups in question, with a *p* < 0.05 considered statistically significant. Data analysis was performed using SPSS Statistics 25 software (IBM Corp., Armonk, NY, USA).

## 3. Results

### 3.1. General Population Characteristics

Overall, 63 patients (of which 62 (98.4%) were male) were included in this study. All patients had self-reported Khat consumption, while 13 (20.6%) had ongoing smoking, and 2 (3.2%) had consumed alcohol. For the entire study population, the PT-INR was 1.2 (1.04–1.35). Transaminitis dominated with ALT 712 (314–1050) IU/L, AST 576 (300–1018) IU/L, GGT 140 (70–305) IU/L, and ALP 162 (134–220) IU/L, respectively. The total bilirubin was 104 (65–225) µmol/L, while the serum albumin was 37 (33–41) g/L. An IgG > 20 g/L was present in 41 (61.5%) patients, while 24 (38.1%) had an AST/ALT > 1, 48 (76.2%) had a hepatocellular (R-factor > 5) injury pattern, 9 (14.3%) had a mixed (R-Factor 2–5) injury pattern, and 6 (9.5%) had a cholestatic (R-factor < 2) injury pattern. The most commonly reported autoantibody was the smooth muscle antibody (SMA), which was positive in 35 (55.6%) patients. LKM1 was equivocal in one (1.6%) of the patients and was, therefore, the least prevalent autoantibody in the patient cohort. Although liver biopsy was performed in 42 (66.7%) of the patients, the results of these biopsies are not available for inclusion in this analysis due to the unavailability of detailed biopsy reports, given that they were performed and interpreted at a different center. A majority of patients, specifically 43 (68.3%) of those included, received treatment with a combination of both azathioprine and steroids. Steroids alone were used in 14 (22.2%) of patients, while 6 (9.5%) patients received no treatment whatsoever. Table 1 illustrates the general characteristics of the included patients.

### 3.2. Biopsy

No difference with regards to age (*p* = 0.351), PT-INR (*p* = 0.646), total bilirubin (0.431), or autoantibody positivity (*p* > 0.5) existed between those who underwent a biopsy (N = 42, 66.7%) and those who did not (N = 21, 33.3%). There was, however, a tendency (*p* = 0.127) towards higher transaminases in the biopsy group, with ALT 870 (364–1100) in the biopsy group versus 459 (209–946) in the non-biopsy group. A similar tendency was observed for AST (*p* = 0.141) and R-factor (*p* = 0.132). There was a tendency towards statistical significance with regards to more combination therapy (*p* = 0.085) being used in the biopsy group (N = 32, 76.2%) versus the non-biopsy group (N = 11, 52.4%). Having no therapy was less prevalent, albeit approaching a statistically significant level (*p* = 0.089) in the biopsy group (N = 2, 4.8%) versus the non-biopsy group (N = 4, 19.0%). Table 2 illustrates the differences between the biopsy versus the non-biopsy groups.

### 3.3. AST/ALT Ratio

Patients with a high AST/ALT had a significantly (*p* = 0.027) higher PT-INR (1.31 (1.10–1.40)) as compared to the PT-INR in those with a lower AST/ALT (1.18 (1.00–1.29)). There was a tendency toward the AST/ALT ratio being associated with older age (*p* = 0.081). There were no differences in positive autoantibodies (*p* > 0.50), IgG levels (*p* = 0.299), or the type of therapy initiated (*p* > 0.50) between the high AST/ALT compared to the low AST/ALT group. Table 3 illustrates the differences in detail.

### 3.4. IgG-Level

Patients with elevated IgG levels of >20 g/L were, albeit non-significantly (*p* = 0.064) younger in age as compared to those with lower IgG levels. The PT-INR was significantly elevated (*p* = 0.002) in patients with higher IgG levels, while albumin was significantly lower (*p* = 0.001) in the high IgG group. There were higher ALTs (albeit non-significant, *p* = 0.187) and ASTs (*p* = 0.009) in the high versus low IgG groups. GGT (*p* = 0.655) and ALP (*p* = 0.634) did not differ between the groups. Autoantibodies did not differ (*p* > 0.05), albeit there was a tendency (*p* = 0.113) towards SMA being more prevalent in the high IgG group. No difference in the R-factor (*p* = 0.478) nor the type of therapy (*p* > 0.05) based on the IgG level was present. Table 4 illustrates the analyses based on the IgG levels in detail.

### 3.5. R-Factor

Patients in the high R-factor group had a tendency (*p* = 0.061) towards elevated PT-INR levels. Bilirubin was significantly elevated (*p* = 0.017) in the high R-factor group, while there was a tendency towards IgG elevation (*p* = 0.110) in the high R-factor group compared to the low R-factor group. No difference (*p* > 0.50) with regard to autoantibody presence existed between both groups. Combined (*p* = 0.057) and steroid-only (*p* = 0.078) therapies were more often prescribed—albeit non-significantly—in the high R-factor group as compared to the low R-factor group. Table 5 illustrates the differences based on the R-factor. Table 5 illustrates the differences as stratified by the R-factor.

## 4. Discussion

In this study, we provide detailed analyses with regard to the laboratory features associated with Khat-induced hepatitis while, at the same time, trying to discern any biochemical patterns that would help shed light on the mechanisms underlying this likely common yet underreported condition. Several interesting observations were evident through the results of our study, hopefully providing context on the pathogenesis of this condition.

The overwhelming majority of participants in this study were male, reflecting the stark gender disparity in Khat consumption, with a clear male predominance in that regard. This also aligns with the well-documented high prevalence of Khat consumption among males in the KSA (the setting of our study) [28], Ethiopia, and Yemen [29,30]. In these regions, lifetime prevalence rates of Khat use are as high as 42% in males compared to just 11% in females [8]. Additionally, evidence suggests that Khat consumption is most common among individuals in their mid-thirties [31], consistent with the age distribution observed in our study. Interestingly, the predominantly AIH-like hepatocellular presentation seen in this population differs significantly from the classic profile of AIH, which typically affects older females with a stronger predisposition to autoimmunity [32]. This discrepancy may point toward a direct Khat-associated effect as the likely cause of the observed injury patterns, mitigating an immune response leading to an AIH-like picture. However, a study from Ethiopia showed no significant difference between Khat users versus non-users with regard to the ANA, SMA, AMA, or IgG levels, which may challenge the aforementioned point raised [33].

No differences were found between the patients when stratified by the AST/ALT ratio. The AST/ALT also acts as a marker to assess for more “Zone III” (central hepatocytes, AST relatively higher) versus “Zone I” (peripheral hepatocytes, ALT relatively higher) predominant injuries, with an elevated AST commonly seen in cases of ischemic hepatitis, especially in the early phase of such injury [23,34]. This is important to note given that Khat is—considering its active substances Cathinone and Cathine—an amphetamine-like substance [35] that may act in a similar pattern, for example, through hypoxic hepatitis, as amphetamine and related substances act when they affect the liver [36,37,38,39]. Such an ischemic injury would cause elevated oxidative stress, which—in the setting of Khat—has already been established on the cellular level with an ameliorative effect of the oxidative state when N-acetyl cysteine (NAC) is administered [40]. Interestingly, NAC has been used to reverse amphetamine-induced hepatic failure on a case report level but not in larger studies [41]. As far as we are aware, however, there are no available reports on the usage of NAC in Khat-induced hepatitis in humans, and we did not use it in any of the patients included in this study. In spite of this, NAC administration is considered generally safe [42] and should be considered in those with severe Khat-induced hepatitis given the mechanism of pathogenesis outlined above, with hopefully future studies to be undertaken with this specific question at hand.

In our study, no significant differences, except for the more extensive medical therapy given to the biopsy group, were present in the characteristics of the patients who underwent liver biopsy versus those who did not, which may, in effect, downplay the importance of performing liver biopsies in patients with known Khat exposure and liver enzyme elevation. However, we must interpret these results with care, given the highly selected patient cohort, the absence of control groups for classic AIH and Khat consumers without liver injury, and the lack of long-term follow-up data. To add more, the difference in the treatment provided in both groups is due to the non-biopsy group usually being unwilling to undergo extensive medical treatment. In our center, we considered liver biopsy in all cases of Khat-induced hepatitis to aid in establishing injury patterns, any underlying advanced hepatic fibrosis or cirrhosis that may suggest the need for extensive follow-up, and the commencement of immunosuppressive treatment where applicable. This liver biopsy-centered approach should remain the standard of care for these patients until reliable scoring systems or biomarkers specifically identifying Khat-induced liver injury are developed in future studies.

The distinction between DILI and AIH can be challenging on liver histology alone without appropriate history details [43], albeit AIH-related hepatic inflammation is plasma-cell dominant, and DILI-induced inflammation involves intracellular cholestasis due to impaired bilirubin secretion from the afflicted hepatocytes [44,45]. Khat-induced hepatitis has been previously reported to have features of lymphocytic and eosinophilic inflammation upon liver biopsy, which is unlike the classic AIH picture described above [17]. In our study, patients with an elevated IgG (>20 g/L) seem to have a larger degree of hepatocyte injury given the higher PT-INR, lower albumin, higher degree of transaminitis, and more elevated bilirubin than those with lower IgG levels. The patients with an elevated IgG were also generally younger in age in our study, which is quite similar to the male phenotype of AIH, which usually affects younger males, albeit in non-comparable populations [45,46]. We, therefore, surmise that the majority of the patients with Khat-induced liver injury have a pattern of liver injury indistinguishable from AIH, also known as drug-induced autoimmune-like hepatitis (DI-ALH) [47,48], and should be treated with immunosuppressants as per the AIH-treatment protocol, especially if there is evidence of AIH-like patterns on the liver biopsy and a simplified AIH score consistent with probable or definite AIH [49], and if the liver function test does not respond to a simple cessation of exposure to Khat. Patients with an IgG > 20 g/L also had, to a larger extent, a higher prevalence of autoantibodies, which—albeit non-significant statistically—signifies further strength to DI-ALH being the major DILI form that Khat induces.

Interestingly, and as per previous reports, the overwhelming injury pattern as per the liver function tests in Khat-induced hepatitis is a hepatocellular one, as over 75% of the patients included in our study had an R-factor > 5. This is—to our knowledge—the first formal assessment of the R-factor in the setting of Khat-induced liver injury, apart from another report with a small number of patients with Khat-induced liver injury showing a mainly hepatocellular pattern [50]. However, in our study, around 14% had a mixed injury pattern, and 10% had a cholestatic pattern. Interestingly, the patients with an R-factor below 5 had less elevated IgG levels, which—albeit non-significant statistically speaking—may suggest a more “bland” DILI pattern without significant autoimmunity in those specific patients. This is further supported by the less aggressive treatment approach in these specific patients with a lower (albeit non-significantly) degree of immunosuppression utilized for these patients as compared to those with a higher R-factor. Patients with a high R-factor were also admittedly “sicker”, given the statistical tendency towards a higher PT-INR and statistically significant bilirubin elevation in those patients. The numbers of patients in the low R-factor group (comprising mixed and cholestatic patterns) were, however, too small to perform a comparison among the three groups.

There are several strengths in our study. First of all, it comprises quite a homogenous population with no underlying viral hepatitis, cirrhosis, or significant alcohol consumption, and thus, the results obtained are quite reassuring and can be attributed specifically to Khat-induced liver injury. In addition, the number of patients is quite large compared to the available reports published on the condition previously. Furthermore, in this study, we provide reference material with regard to the laboratory values to be expected in patients with Khat-induced liver injury, with a specific focus on the R-factor, which, to our knowledge, has not been extensively studied in this specific population. However, the R-factor itself is limited given that it does not take into consideration the deficit in liver function occurring due to DILI and should be used in conjunction with the known markers of liver function, such as albumin and PT-INR.

However, there are several limitations that need to be addressed. The most important limitation of our study is the unavailability of biopsy reports for the included patients. This limits the interpretation of the available data and would have enriched the analysis further if those details were available; for example, through the calculation of the simplified AIH score in those patients with an available biopsy report, which would provide a reference for future research. Another limitation is the absence of detailed ultrasound data, which would have been valuable to correlate to the available laboratory data in this study. In addition, the absence of significant outcome data with regard to mortality or progression to cirrhosis is another limitation. A significant limitation of this study is the absence of a well-defined control group, which is crucial for drawing stronger conclusions. The lack of a comparison group with classic AIH, a second group of Khat users without liver injury, and a third group of healthy non-Khat consumers with normal liver function tests prevents a comprehensive evaluation of Khat-induced liver injury. Including these controls would have allowed for a refined analysis between classic AIH and Khat-induced liver dysfunction, as well as a clearer understanding of its true impact compared to unaffected individuals. Importantly, another weakness is the absence of anthropometric data, especially with regard to the body mass index (BMI) of those included in this study. This is in light of the evidence pointing towards a higher degree of undernutrition in Khat consumers which may, in effect, act as a confounding factor to interpreting the data [31]. Another weakness is the absence of detailed background data with regard to the presence of underlying diabetes, given the prevalence of diabetes in our population [51] and the association of diabetes and metabolic factors with undiagnosed metabolic liver disease [52]. Another limitation is the absence of a formal assessment of alcohol intake with laboratory markers, such as phosphatidylethanol (PETh) or carbohydrate-deficient transferrin (CDT), as occult consumption may have been missed or not reported when only resorting to history-taking for this specific information [53]. Alcohol consumption is—generally speaking—socially unacceptable [54] and is, therefore, most likely underreported as compared to the likely more normative use of Khat in our society [55]. This could even affect the interpretation of the AST/ALT ratio data, as this ratio is significantly influenced by alcohol consumption and alcohol-related liver disease [56].

## 5. Conclusions

Khat-induced liver injury seems to be overwhelmingly AIH-like in nature, albeit certain patients may demonstrate liver injury patterns similar to mixed/cholestatic DILI and/or ischemic hepatitis. Further research into novel therapies such as NAC (based on previous preclinical data), long-term progression of the patients afflicted with Khat-induced liver injury to cirrhosis, and a correlation of laboratory values with liver biopsy findings is a necessary next step in the research related to this likely underreported condition.

## Figures and Tables

**Table 1:** The general population characteristics.

Patient Characteristics	Data (% or IQR)
Gender (Male)	62 (98.4)
Age (Years)	35 (29–41)
Smoking (active)	13 (20.6)
Alcohol consumption	2 (3.2)
PT-INR ratio	1.2 (1.04–1.35)
ALT (IU/L)	712 (314–1050)
AST (IU/L)	576 (300–1018)
GGT (IU/L)	140 (70–305)
ALP (IU/L)	162 (134–220)
Total bilirubin (µmol/L)	104 (65–225)
Albumin (g/L)	37 (33–41)
IgG levels (g/L) *	24.0 (18.0–30.1)
Antinuclear antibodies (ANA)	30 (47.6)
Smooth muscle antibodies (SMA)	35 (55.6)
Antimitochondrial antibodies (AMA)	3 (4.8)
AST/ALT ratio **	0.893 (0.622–1.163)
R-factor ***	13.0 (6.58–20.6)

Notes: * 41 (61.5%) with IgG > 20 g/L; ** 24 (38.1%) with AST/ALT ratio > 1; *** 48 (76.2%) with R-factor > 5; 9 (14.3%) with R-factor 2–5; and 6 (9.5%) with R-factor < 2.

**Table 2:** Characteristics of those who underwent biopsy versus those who did not.

Patient Characteristics	Biopsy (N = 42)	No Biopsy (N = 21)	*p*-Value
Age (Years)	35 (27.5–41.5)	38 (33–40.5)	0.351
Smoking (active)	9 (21.4)	4 (19.0)	1.000
PT-INR ratio	1.2 (1.04–1.37)	1.20 (1.03–1.31)	0.646
ALT (IU/L)	870 (364–1100)	459 (209–946)	0.127
AST (IU/L)	692 (352–1033)	513 (145–850)	0.141
GGT (IU/L)	162 (80–295)	103 (54–595)	0.848
ALP (IU/L)	166 (138–222)	149 (124–207)	0.326
Total bilirubin (µmol/L)	123 (70–226)	92 (48–233.5)	0.431
Albumin (g/L)	37 (33–40)	38 (35–45)	0.190
IgG levels (g/L)	24 (18.5–32.1)	21.8 (17.0–29.8)	0.355
Antinuclear antibodies (ANA)	19 (45.2)	11 (52.4)	0.606
Smooth muscle antibodies (SMA)	22 (52.4)	13 (61.9)	0.593
Antimitochondrial antibodies (AMA)	3 (7.1)	0 (0)	0.545
AST/ALT ratio	0.884 (0.641–1.21)	0.923 (0.497–1.14)	0.649
R-factor	15.0 (7.85–20.9)	9.29 (2.97–19.2)	0.132
Combination therapy	32 (76.2)	11 (52.4)	0.085
Steroid therapy only	8 (19.0)	6 (28.6)	0.522
No therapy	2 (4.8)	4 (19.0)	0.089

**Table 3:** Characteristics stratified by the AST/ALT ratio.

Patient Characteristics	AST/ALT > 1 (N = 24)	AST/ALT < 1 (N = 39)	*p*-Value
Age (Years)	40.5 (30.8–44.0)	35.0 (29.0–39.0)	0.081
Smoking (active)	4 (16.7)	9 (23.1)	0.750
PT-INR ratio	1.31 (1.10–1.40)	1.18 (1.00–1.29)	0.027
GGT (IU/L)	113 (68.5–281.5)	174 (70.0–353)	0.651
ALP (IU/L)	166 (109.5–219.5)	152 (142–220)	0.772
Total bilirubin (µmol/L)	139 (81.8–240)	94.0 (60.0–188.0)	0.208
Albumin (g/L)	37.0 (29.3–41.8)	37.0 (34.6–41.0)	0.534
IgG levels (g/L)	26.0 (17.3–32.8)	22.8 (18.0–29.7)	0.299
Antinuclear antibodies (ANA)	11 (45.8)	19 (48.7)	1.000
Smooth muscle antibodies (SMA)	11 (45.8)	24 (61.5)	0.298
Antimitochondrial antibodies (AMA)	1 (4.2)	2 (5.1)	1.000
R-factor	9.90 (4.46–17.7)	14.9 (6.79–21.7)	0.182
Combination therapy	17 (70.8)	26 (66.7)	0.787
Steroid therapy only	5 (20.8)	9 (23.1)	1.000
No therapy	2 (8.3)	4 (10.3)	1.000

**Table 4:** The characteristics stratified by IgG level.

Patient Characteristics	IgG > 20 g/L (N = 41)	IgG < 20 g/L (N = 22)	*p*-Value
Age (Years)	34.0 (27.0–41.0)	38.5 (34.8–43.3)	0.064
Smoking (active)	8 (19.5)	5 (22.7)	0.755
PT-INR-ratio	1.27 (1.09–1.42)	1.07 (1.00–1.23)	0.002
ALT (IU/L)	804 (450–1049)	437 (185–1109)	0.187
AST (IU/L)	731 (384–1055)	388 (104–780)	0.009
GGT (IU/L)	140 (56–299)	128 (80.5–375)	0.655
ALP (IU/L)	163 (135–218)	150 (127–221)	0.634
Total bilirubin (µmol/L)	127 (73.5–243)	90 (37.5–158)	0.048
Albumin (g/L)	35 (29.3–39.0)	41.0 (36.9–44.0)	0.001
Antinuclear antibodies (ANA)	22 (53.7)	8 (36.4)	0.290
Smooth muscle antibodies (SMA)	26 (63.4)	9 (40.9)	0.113
Antimitochondrial antibodies (AMA)	3 (7.3)	0 (0)	0.546
R-factor	14.3 (8.28–19.2)	9.90 (2.97–21.3)	0.478
Combination therapy	29 (70.7)	14 (63.6)	0.582
Steroid therapy only	8 (19.5)	6 (27.3)	0.543
No therapy	4 (9.8)	2 (9.1)	1.000

**Table 5:** Characteristics stratified by the R-factor.

Patient Characteristics	R-Factor > 5 (N = 48)	R-Factor < 5 (N = 15)	*p*-Value
Age (Years)	35.0 (29.0–41.0)	39.0 (33.0–41.0)	0.459
Smoking (active)	10 (20.8)	3 (20.0)	1.000
PT-INR ratio	1.23 (1.07–1.40)	1.07 (1.00–1.32)	0.061
ALT (IU/L)	933 (574–1145)	170 (102–272)	<0.001
AST (IU/L)	812 (486–1061)	109 (80.0–300)	<0.001
GGT (IU/L)	127 (57–290)	190 (83.0–762)	0.112
ALP (IU/L)	150 (124–207)	224 (167–527)	0.002
Total bilirubin (µmol/L)	132 (76.8–240)	68.0 (21.0–100)	0.017
Albumin (g/L)	36.5 (33.0–40.0)	41.0 (34.0–42.0)	0.297
IgG (g/L)	24.1 (18.6–31.5)	19.6 (16.5–25.0)	0.110
Antinuclear antibodies (ANA)	22 (45.8)	8 (53.3)	0.769
Smooth muscle antibodies (SMA)	27 (56.3)	8 (53.3)	1.000
Antimitochondrial antibodies (AMA)	3 (6.3)	0 (0)	1.000
Combination therapy	36 (75.0)	7 (46.7)	0.057
Steroid therapy only	8 (16.7)	6 (40.0)	0.078
No therapy	4 (8.3)	2 (13.3)	0.622

## Data Availability

The data presented in this study are available on request from the corresponding author.
